# Structured group education programme and accompanying mHealth intervention to promote physical activity in women with a history of gestational diabetes (*Baby Steps*): 4‐year follow‐up of a randomised controlled trial

**DOI:** 10.1111/dom.70199

**Published:** 2025-10-21

**Authors:** Patrick J. Highton, Mark P. Funnell, Aikaterina Tziannou, Alex V. Rowlands, Nithya Sukumar, Clare L. Gillies, Laura J. Gray, Tom Yates, Melanie J. Davies, Ponnusamy Saravanan, Kamlesh Khunti

**Affiliations:** ^1^ Diabetes Research Centre, College of Life Sciences University of Leicester Leicester UK; ^2^ NIHR Applied Research Collaboration East Midlands, Diabetes Research Centre University of Leicester Leicester UK; ^3^ Leicester Real World Evidence Unit, Diabetes Research Centre University of Leicester, Leicester General Hospital Leicester UK; ^4^ Alliance for Research in Exercise, Nutrition and Activity (ARENA), UniSA Allied Health and Human Performance University of South Australia Adelaide Australia; ^5^ Warwick Applied Health, Warwick Medical School, Gibbet Hill University of Warwick Coventry UK; ^6^ Department of Diabetes, Endocrinology and Metabolism George Eliot Hospital Nuneaton UK; ^7^ Department of Population Health Sciences University of Leicester Leicester UK; ^8^ Leicester British Heart Foundation Centre of Research Excellence University of Leicester Leicester UK; ^9^ Warwick Centre for Global Health, Warwick Medical School University of Warwick Coventry UK

**Keywords:** exercise intervention, gestational diabetes, glycaemic control, randomised trial

## 1 BACKGROUND

Gestational diabetes mellitus (GDM) is associated with a substantially increased risk of developing type 2 diabetes mellitus (T2DM), with up to 50% of women progressing to prediabetes or T2DM within 10 years postnatal.^1^ Despite guidance from the National Institute for Health and Care Excellence recommending postnatal lifestyle advice and monitoring,^2^ long‐term engagement remains low.^3^ We developed and evaluated the *Baby Steps* programme, a group‐based education intervention and mHealth application, designed to promote physical activity (PA) in a multi‐ethnic UK cohort of women with recent GDM.^4^

In our previously published randomised controlled trial (RCT), the *Baby Steps* intervention led to improvements in self‐efficacy for exercise, quality of life, and anxiety, and the primary outcome of objectively measured PA showed a non‐significant but potentially clinically significant between‐group difference at 12 months (equivalent to an additional ~500 steps/day).^5^ The intervention was shown to be cost‐effective and has been implemented into routine care internationally.

Few studies have reported long‐term outcomes following lifestyle interventions in women with prior GDM. In this study, we report 4‐year follow‐up data from the *Baby Steps* trial cohort,^5^ assessing the long‐term impact of the intervention on behavioural, psychosocial, and clinical outcomes.

## 2 METHODS

The original RCT protocol and outcomes were reported previously.^4^, ^5^ This follow‐up study collected data at 48 months post‐baseline from participants in the original trial, conducted under amended ethical approval (16/EM/0488). Data were collected between July 2021 and November 2022.

### 2.1 Participants

Eligible participants were women aged ≥18 years who had previously enrolled in the *Baby Steps* RCT. They were identified through GDM registers at two hospitals in England (Leicester and Nuneaton), had a GDM diagnosis during any pregnancy within the past 5 years, and had consented to a 48‐month follow‐up. Full eligibility criteria are detailed elsewhere.^5^

### 2.2 Procedure

Participants in the original RCT were randomised to the intervention or control group. The *Baby Steps* intervention, described in detail elsewhere,^4^ comprised two, 3‐h group education sessions based on the Let's Prevent Diabetes programme,^6^ and an mHealth component.^4^ The group sessions were designed to increase PA and overall lifestyle management, while the mHealth component was a mobile web application comprised of interactive information via video animations, expert videos, activities, quizzes, activity “leader boards” and follow‐up booster sessions designed to complement the group education sessions and support increased PA.

### 2.3 Outcomes

PA data were collected using a GENEActiv accelerometer (Activinsights Ltd.), worn on the non‐dominant wrist for 8 days. The primary PA outcome was change in daily average acceleration in milligravitational units (mg). Data processing mimicked the original trial,^5^ including the approximation of mg data into steps/day via triangulation of data sources as explained by Rowlands et al.^7^ Questionnaires, completed postally, included the Jenkins Self‐Efficacy‐for‐Exercise‐Scale,^8^ the Hospital Anxiety and Depression Scale (HADS),^9^ and health‐related quality of life (EQ‐5D‐5L).^10^ Glycated haemoglobin (HbA1c) data were obtained from routine healthcare records between 12 and 48 months, where available.

### 2.4 Statistical Analyses

All data presented (including 12‐month data from the original trial) relate to the follow‐up cohort only. Data were analysed using linear mixed regression models with adjustment for age, ethnicity, body mass index (BMI), and baseline outcome values. For the assessment of PA, models were additionally adjusted for change from baseline in accelerometer wear days. Fixed effects included baseline data, randomisation group, time point, and group‐by‐time interaction. Participant‐level random effects were included to account for repeated measurements. Results are presented as adjusted mean differences between intervention and control groups with corresponding 95% confidence intervals. Fisher's exact test was used to assess statistical significance due to small cell counts. Development of T2DM, defined as HbA1c ≥6.5% at any timepoint between 12 and 48 months, was assessed as a binary outcome. Risk ratios were calculated using unconditional maximum likelihood estimation. Statistical significance was defined as *p* < 0.05.

## 3 RESULTS

### 3.1 Trial flow

Of the original trial cohort, 285 (97.3%) participants were eligible for follow‐up at 48 months. Among these, 136 (47.7%) either did not respond or were unwilling to participate in the follow‐up study. A total of 149 (52.3%) participants (*n* = 79 control, *n* = 70 intervention) returned completed questionnaires, and of these, 100 (67.1%) returned accelerometers. HbA1c data were available for 52 (34.9%) participants (see Data S1, Supporting Information, for a trial flow diagram). Mean age was 35.2 years, BMI was 28.3 m/kg^2^, and 68% of participants identified as White ethnicity. No significant differences in sociodemographic or baseline PA variables were found between follow‐up participants and non‐participants from the original trial. Mean change in PA from the original 12‐month trial was not significantly different between follow‐up participants and non‐participants (*p* = 0.84), indicating that responders to the intervention were not disproportionately represented in this follow‐up cohort.

### 3.2 Physical activity

Eighty participants returned accelerometers (*n* = 41 control, *n* = 39 intervention) that contained valid data (≥1 day of data at baseline, 12 and 48 months). No significant difference in daily PA was observed at 12 (*p* = 0.53) or 48 months (*p* = 0.10) between groups (Table 1). At 48 months, the intervention group showed a non‐significant between‐group difference of 1.94 mg (95% confidence interval [CI]: −0.36 to 4.25) in daily average acceleration, equivalent to approximately 1000 additional steps/day.

**TABLE 1:** Mean change from baseline and adjusted mean difference at follow‐up for intervention and control groups for PA (expressed in milligravitational units, mg), at 12 and 48 months.

Follow‐up^a^	Mean change from baseline (mg)		Adjusted difference at follow up (mg)^b^	
	Control (*n* = 41)	Intervention (*n* = 39)	Coefficient (95% CI)	*p*‐Value
12 months	0.15	1.13	0.72 (−1.58, 3.03)	0.53
48 months	−0.81	1.41	1.94 (−0.36, 4.25)	0.10

Abbreviations: BMI, body mass index; CI, confidence interval; PA, physical activity.

^a^ Participants with at least 1 day of valid accelerometer data at baseline, 12, and 48 months.

^b^ Intervention − Control comparison. Adjusted for: age (<30, ≥30), ethnicity, BMI, baseline PA value, time (0–12, 0–48), change from baseline in accelerometer wear days, time * randomisation group interaction.

### 3.3 Questionnaires

Questionnaire completion rates were similar in both groups (Table 2). A significant improvement in self‐efficacy‐for‐exercise (SEE) was observed at 12 months in the intervention group (6.28 [95% CI 0.38, 12.19], *p* = 0.04). No other significant differences were found for SEE, anxiety and depression (HADS), or quality of life (EQ‐5D‐5L) at either 12 or 48 months (*p* ≥ 0.06; Table 2).

**TABLE 2:** Mean change from baseline and adjusted mean difference at follow‐up for intervention and control groups for questionnaire‐based outcomes, including SEE, anxiety and depression (HADS), and health‐related quality of life (EQ‐5D‐5L), at 12 and 48 months.

Questionnaire	Follow‐up (12 and 48 months)	Number of participants		Mean change from baseline		Adjusted difference at follow up^a^	
		Control	Intervention	Control	Intervention	Coefficient (95% CI)	*p*‐Value
SEE	SEE‐12	69	61	−3.26	3.13	6.28 (0.38, 12.19)	0.04*
	SEE‐48	76	66	−4.03	−1.94	1.95 (−3.76, 7.65)	0.5
HADS	Anxiety‐12	70	65	0.46	−0.88	−0.9 (−2.04 0.23)	0.12
	Anxiety‐48	79	70	1.15	1.26	0.42 (−0.67, 1.50)	0.45
	Depression‐12	70	65	0.23	−0.40	−0.57 (−1.71, 0.57)	0.32
	Depression‐48	77	69	0.79	1.20	0.40 (−0.70, 1.50)	0.47
EuroQoL (EQ‐5D‐5L)	Index score‐12	70	65	−0.05	−0.03	0.02 (−0.03, 0.07)	0.38
	Index score‐48	71	65	−0.08	−0.07	0.02 (−0.03 0.07)	0.5
	VAS scale‐12	71	65	−1.00	4.48	4.83 (−0.22, 9.88)	0.06
	VAS scale‐48	78	69	−3.68	0.39	3.29 (−1.58, 8.16)	0.18

^a^ Intervention – Control comparison. Adjusted for: age (<30, ≥30), ethnicity, BMI, baseline outcome value, time (0–12, 0–48), time*randomisation group interaction.

* *p* < 0.05

### 3.4 Glycated haemoglobin

Of the 52 participants (*n* = 30 control, *n* = 22 intervention) with available HbA1c data at 48 months (mean follow‐up was 3.08 years), no significant effect of the intervention was observed (*p* = 0.41; Data S2). For the assessment of T2DM development, 102 participants (*n* = 54 control, *n* = 48 intervention) had HbA1c data available between 12 and 48 months. During this period, 10 participants (9.8%) developed T2DM; 6 (11.1%) in control and 4 (8.3%) in intervention. The risk ratio for developing T2DM was 0.75 (95% CI: 0.23–2.50, *p* = 0.75).

## 4 CONCLUSIONS

No significant differences in device‐measured PA were observed in this follow‐up cohort. However, at 48 months, a non‐significant between‐group difference of 1.94 mg in daily average acceleration, equivalent to ~1000 additional steps/day, was observed. This is consistent with, and even greater than, the original trial findings, which showed a non‐significant increase of ~500 steps/day at 12 months.^5^ Although this may represent a clinically relevant difference in PA levels, it was not statistically significant and was based on a relatively low follow‐up rate at 48 months. Accordingly, this finding should be considered exploratory rather than confirmatory.

No significant effects were observed for anxiety, depression, quality of life, or HbA1c at 48 months, though the interpretation of HbA1c was limited by the small number of participants with available data. The limited availability of long‐term HbA1c data highlights a concerning gap in routine postnatal diabetes monitoring for women with prior GDM, potentially impacting the timely diagnosis of T2DM in this high‐risk population.^3^

Most lifestyle interventions targeting women with a history of GDM have focused primarily on postpartum weight loss,^11^ often using self‐reported PA as a secondary outcome. The largest trials to date, Mothers After Gestational Diabetes in Australia (MAGDA)^12^ and Gestational Diabetes' Effects on Moms (GEM),^13^ delivered interventions within the first year postpartum and relied on self‐reported PA measures. GEM reported a small increase in vigorous PA (15 min/week), while MAGDA found no significant differences in achieving activity goals.^12^, ^13^ A smaller study that utilised text messaging and pedometer‐based measurement of physical activity found a non‐significant trend towards increased activity,^14^ however, very few studies have objectively measured physical activity. These findings reflect a broader limitation in the evidence base; specifically, the reliance on self‐reported measures and a lack of long‐term, objective physical activity data.

The mHealth application had low initial uptake; only 66% of those who attended group sessions (representing 54% of all intervention participants) registered for the application, and engagement with its features was limited.^5^ This pattern is not unique; in MAGDA, 66% of participants attended the initial face‐to‐face intervention session, only 53% attended one or more of five group sessions, and 34% had no exposure to the intervention.^12^ In GEM, which used a telephone‐based approach, only 50% of participants completed at least one call.^13^ The low engagement levels highlight a consistent challenge in participation in postpartum and mHealth interventions. Strategies to improve retention and engagement with mHealth interventions in this population are needed and could include: (1) personalisation of content to align with individual goals^15^; (2) incorporating gamification and deeper interactivity^16^; (3) integration with routine healthcare, for instance primary care follow‐up visits, and (4) embedding behaviour change techniques into the intervention's functionality.^17^

Although this study benefited from the use of device‐measured physical activity and a long‐term follow‐up period, the observed attrition rate (149 of 285 participants) was substantial, though not unexpected given the extended follow‐up duration. The limited availability of HbA1c data restricted interpretation of glycaemic outcomes. Furthermore, it was not possible to determine which element(s) of the intervention were responsible for the observed changes. Future research should incorporate process evaluations to address this limitation.

These findings evidence the challenge of the long‐term impact of a GDM‐specific lifestyle intervention. Though non‐significant, the increase in PA at 48 months, equivalent to ~1000 steps/day, could have meaningful clinical benefits. Future interventions should incorporate components for ongoing engagement, behaviour change models, and better integration with routine care to translate short‐term benefits into lasting behavioural change and positive clinical outcomes.

## AUTHOR CONTRIBUTIONS

Patrick J. Highton: data curation, analysis, writing – original draft, project administration. Mark P. Funnell: analysis, writing – original draft, project administration. Aikaterina Tziannou: analysis, writing – original draft. Alex V. Rowlands, Nithya Sukumar, Melanie J. Davies, Tom Yates: writing– review and editing. Ponnusamy Saravanan: conceptualisation, data curation, writing – review and editing. Clare L. Gillies, Laura J. Gray, Kamlesh Khunti: conceptualisation, supervision, writing – review and editing.

## CONFLICT OF INTEREST STATEMENT

Kamlesh Khunti has acted as a consultant, speaker, or received grants for investigator‐initiated studies for Astra Zeneca, Bayer, Novartis, Novo Nordisk, Sanofi‐Aventis, Lilly, and Merck Sharp & Dohme, Boehringer Ingelheim, Oramed Pharmaceuticals, Pfizer, Roche, Daiichi‐Sankyo, and Applied Therapeutics. Melanie J. Davies has acted as a consultant/advisor and speaker for Eli Lilly, Novo Nordisk, and Sanofi, has attended advisory boards for AbbVie, Amgen, AstraZeneca, Biomea Fusion, Carmot/Roche, Daewoong Pharmaceutical, Sanofi, Zealand Pharma, Regeneron, GSK, and EktaH, and as a speaker for AstraZeneca, Boehringer Ingelheim, and Zuellig Pharma. Melanie J. Davies has received grants from AstraZeneca, Boehringer Ingelheim, and Novo Nordisk. No other authors report conflicts of interest.

## Supporting information


**Data S1:** Trial flow diagram.
**Data S2:** Glycated haemoglobin (HbA1c).
**Data S3:** Visual abstract.
**Table S1:** Mean change from baseline and adjusted mean difference at follow‐up for intervention and control groups for HbA1c, at 12 and 48 months.

## Data Availability

The data that support the findings of this study are available from the corresponding author upon reasonable request.
