# Supplementary material for: Structured group education programme and accompanying mHealth intervention to promote physical activity in women with a history of gestational diabetes (Baby Steps): 4‐year follow‐up of a randomised controlled trial
Source: Diabetes Obes Metab. 2025 Oct 21;28(1):754–8. doi: 10.1111/dom.70199 (PMC12673440; doi:10.1111/dom.70199)

**Supplementary Materials**

Highton et al. (2025) Structured group education programme and accompanying mHealth intervention to promote physical activity in women with a history of gestational diabetes (*Baby Steps*): 4-year follow-up of a randomised controlled trial

**Supplementary Material 1: Trial Flow Diagram**

N=285 original trial participants eligible for follow-up and contacted

N=149 participated in follow-up study

N=136 either did not respond or were unwilling to participate

N=149 completed questionnaires (completion rates varied across questionnaires, see Table 2)

N=100 returned accelerometers, N=52 had valid available HbA1c data at 48 months

N=49 did not return accelerometers
N=97 did not have valid available HbA1c data at 48 months

**Supplementary Material 2:** Glycated Haemoglobin **(HbA1c)**

**Supplementary Table 1.** Mean change from baseline and adjusted mean difference at follow-up for intervention and control groups for HbA1c, at 12 and 48 months.

| **Follow-up** | **Mean change from baseline (%)** | | **Adjusted difference at follow up† (%)** | |
| --- | --- | --- | --- | --- |
|  | **Control (n=30)** | **Intervention (n=22)** | **Coefficient (95% CI)** | ***P* -value** |
| **12 months** | -0.03 | -0.46 | - 0.07 (-3.10, 2.97) | 0.96 |
| **48 months** | 1.60 | 2.50 | 1.25 (-1.78, 4.29) | 0.41 |

† = Intervention - Control comparison. Adjusted for: age (<30, >=30), ethnicity, BMI, baseline HbA1c, time (0-12, 0-48), time*randomisation group interaction. Mean follow up time from 12 months to 48 months was 3.08 years.

**Supplementary Material 3: Visual Abstract**


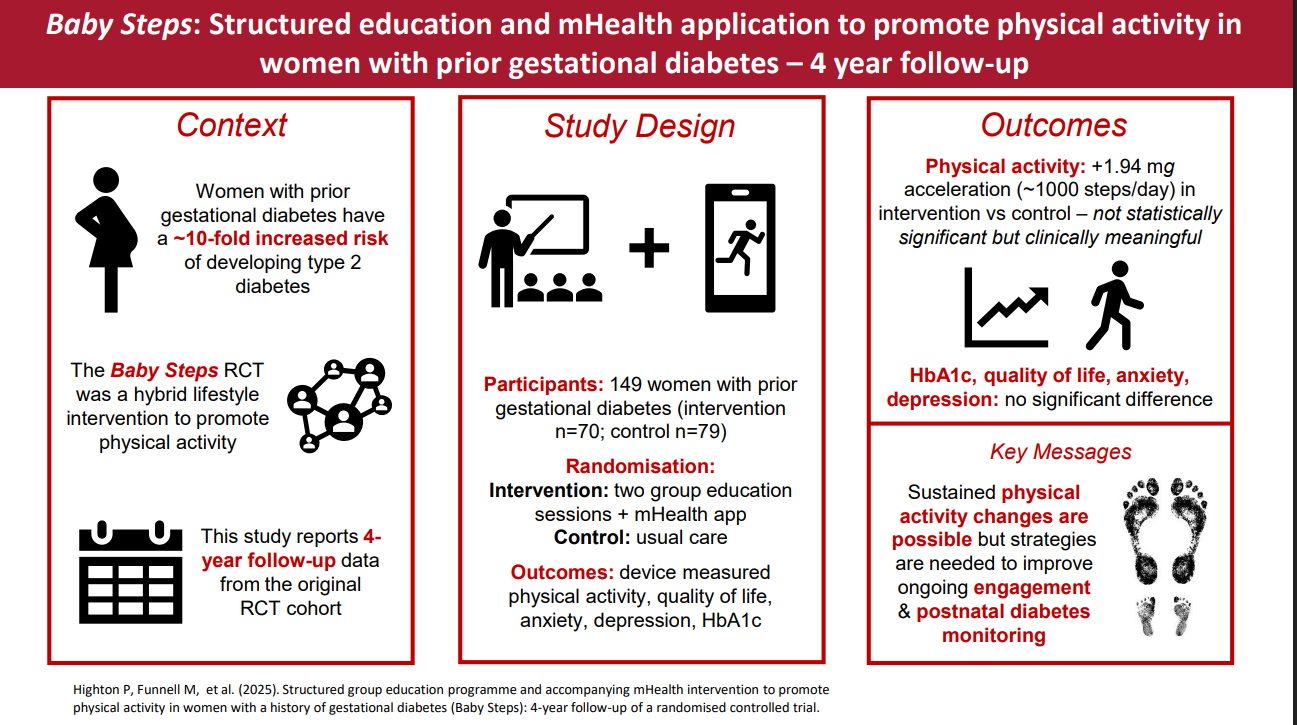

Supplement: Supplementary file 1 — Data S1: Trial flow diagram. Data S2: Glycated haemoglobin (HbA1c). Data S3: Visual abstract. Table S1: Mean change from baseline and adjusted mean difference at follow‐up for intervention and control groups for HbA1c, at 12 and 48 months. [file DOM-28-754-s001.docx]
